# Subacute stent thrombosis with spontaneously resolved secondary thrombi in paroxysmal nocturnal hemoglobinuria: a case report

**DOI:** 10.1186/s12872-022-02850-z

**Published:** 2022-09-12

**Authors:** Hiroshi Kawahara, Nobuhide Watanabe, Akihiro Endo, Hiroyuki Yoshitomi, Kazuaki Tanabe

**Affiliations:** 1 Division of Cardiology, Faculty of Medicine, Shimane University, 89-1 Enya-cho, Izumo, 693-8501 Japan; 2 Clinical Laboratory Division, Faculty of Medicine, Shimane University, 89-1 Enya-cho, Izumo, 693-8501 Japan

**Keywords:** Paroxysmal nocturnal hemoglobinuria, Percutaneous coronary intervention, Stent thrombosis, Antithrombotic therapy, Case report

## Abstract

**Background:**

Stent thrombosis (ST) is a serious complication; however, a method to prevent ST in patients with thrombophilic diseases has not been established.

**Case presentation:**

We report a case of subacute ST in a patient with paroxysmal nocturnal hemoglobinuria (PNH) who was receiving continuous heparin treatment in addition to the usual dual antiplatelet therapy for contrast defects at the proximal site of the occluded right coronary artery and the proximal site of the left circumflex artery. Despite the resolution of thrombi in secondary lesions, subacute ST occurred. After percutaneous coronary intervention for ST, triple therapy, including oral anticoagulation for PNH-related thrombosis, was initiated. The patient subsequently underwent craniotomy hematoma removal for hemorrhagic cerebral infarction.

**Conclusions:**

Reported cases of ST in patients with PNH are very few, and this case adds evidence with respect to antithrombotic therapy in patients with thrombotic tendencies. Both thrombosis and bleeding should be considered when administering antithrombotic therapy to patients with thrombotic diseases. If there are specific treatments for thrombophilic diseases, they should be initiated early.

## Background

Stent thrombosis (ST) is a fatal complication associated with a mortality rate of 5–45% and occurs in 0.6% of patients with acute coronary syndromes, even with intracoronary imaging techniques [1]. High platelet counts and other factors are reported to be risk factors for ST [2]; however, the association of thrombogenic diseases with ST is unclear. Paroxysmal nocturnal hemoglobinuria (PNH) occurs because of an acquired mutation of the phosphatidylinositol glycan class A (*PIGA*) gene, and thrombosis should be considered a complication [3]. Reports on ST in PNH are very few. Herein, we report about a patient with PNH who developed ST despite the resolution of thrombi in secondary lesions due to continuous heparin use and conventional dual antiplatelet therapy (DAPT), followed by craniotomy hematoma removal for a hemorrhagic cerebral infarction. This case adds to the evidence for antithrombotic therapy after drug-eluting stent implantation in patients with PNH.

## Case presentation

A 73-year-old man with myelodysplastic syndrome for 13 years was diagnosed with PNH 2 years ago based on hemolysis, a negative direct Coombs test, and PNH erythrocyte and granulocyte concentrations of 14.6% and 43.1%, respectively.

**Fig. 1:**
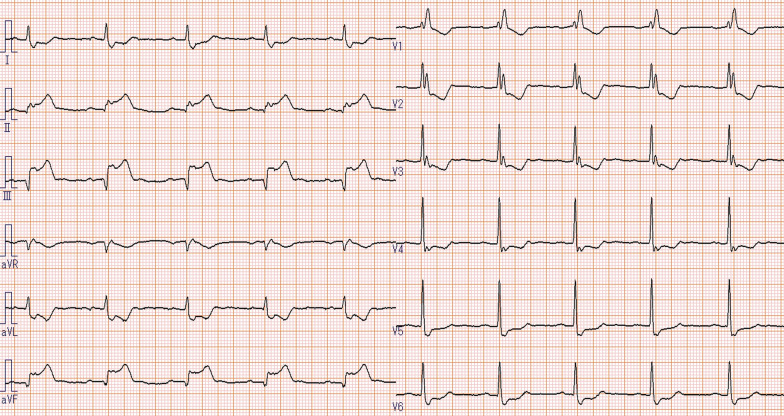
Electrocardiogram from the emergency department. Acute myocardial infarction in the right coronary artery territory is suspected

**Fig. 2:**
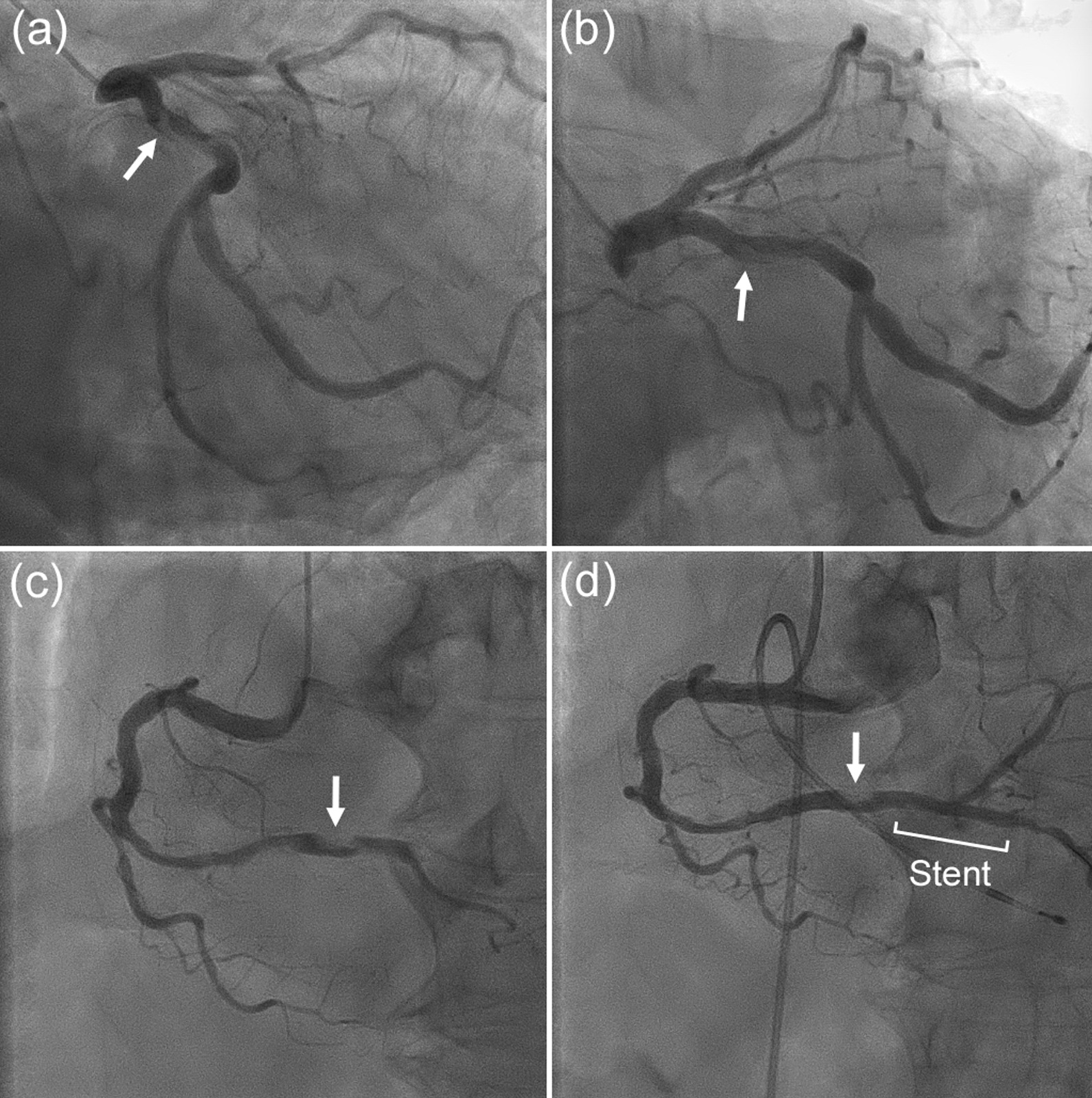
**a**, **b** Right anterior oblique (RAO) and left anterior oblique (LAO) caudal views of the left coronary artery (LCA) at the initial acute myocardial infarction (AMI). Contrast defects were detected at the proximal site of the left coronary artery (white arrows). **c**, **d** The AMI lesion is in the right coronary artery (RCA). The LAO-cranial view of the RCA before percutaneous coronary intervention (PCI) and after an everolimus-eluting stent (2.75 × 24 mm) was implanted. A contrast defect proximal to the lesion in the right coronary artery is noted (white arrows)

**Fig. 3:**
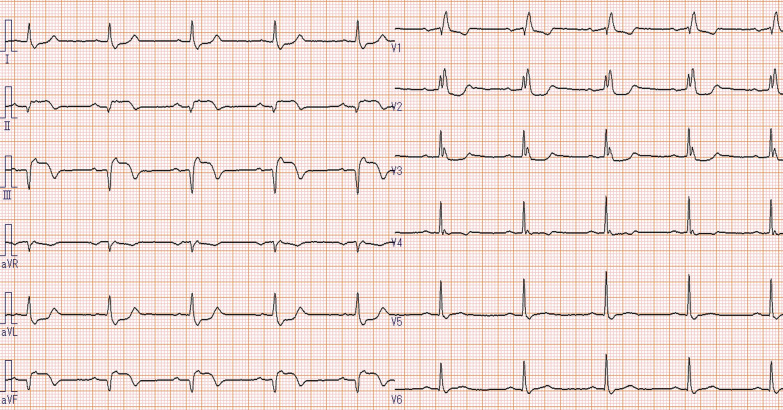
Electrocardiogram of chest pain 2 days after the initial PCI. The right coronary artery appears to be occluded

**Fig. 4:**
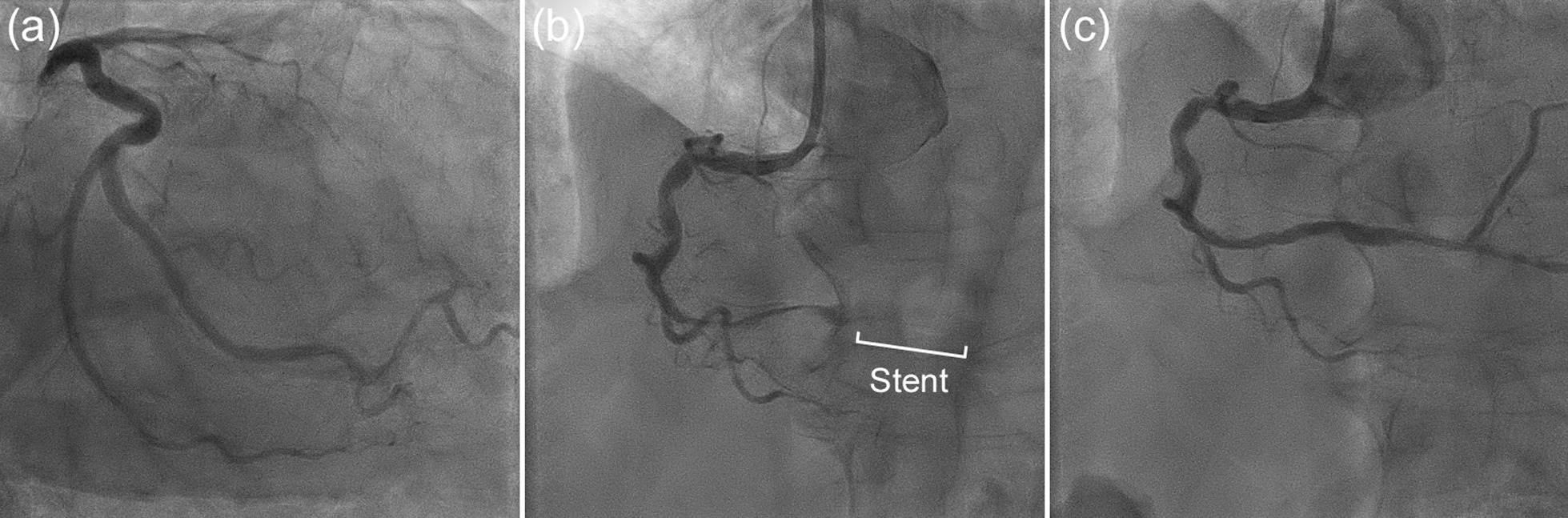
**a** RAO-caudal view of the LCA when stent thrombosis (ST) occurred 2 days after the first PCI. The contrast defect observed in the previous coronary angiography had improved. **b** This is the image of the ST in the LAO-cranial view of the RCA. **c** The second PCI was performed with intra-aortic balloon pumping, thrombus aspiration, and balloon dilatation. The improvement in blood flow in the occlusion was confirmed in the LAO-cranial view of the RCA

The patient presented to the emergency department of our hospital with chest pain, and electrocardiography (ECG) revealed an acute myocardial infarction (AMI) (Fig. 1). Transthoracic echocardiography detected decreased wall motion in the inferior wall region. Emergency coronary angiography (CAG) showed contrast defects at the proximal site of the occluded right coronary artery (RCA) and the proximal site of the left circumflex artery (LCX) (Fig. 2a, b, and c). Aspirin (200 mg) was administered in the emergency department, and clopidogrel 300 mg (Japanese loading dose) was added at the start of percutaneous coronary intervention (PCI). An everolimus-eluting stent (2.75 × 24 mm) was deployed from the distal RCA to the posterior descending artery (Fig. 2d). Heparin was injected to exceed 250 s of activated clotting time (ACT) during PCI, and a sufficiently dilated lumen without malapposition or edge dissection was confirmed using intravascular ultrasound (IVUS). The maintenance dose of DAPT was aspirin 100 mg and clopidogrel 75 mg daily. Continuous heparin was administered to treat thrombi from secondary lesions after PCI, and the heparin dose was gradually increased to 25,000 units/day based on activated partial thromboplastin time (APTT). Two days later, the patient developed chest pain again, and ECG revealed AMI of the inferior wall (Fig. 3); therefore, an emergency CAG was performed. Despite resolution of the defect at the LCX (Fig. 4a), subacute ST occurred in the treated area of the RCA (Fig. 4b). Although the patient had a normal sinus rhythm and was not in shock when the stent thrombosis occurred, intra-aortic balloon pumping (IABP) was implanted to increase coronary blood flow to prevent new thrombus formation and because ST has a high mortality rate, followed by thrombus aspiration, and balloon dilatation were performed during the second PCI (Fig. 4c). As the final IVUS during the initial PCI confirmed that there was no issue with stent implantation, IVUS was not performed during PCI for stent thrombosis. Considering the possibility that the patient maybe a poor metabolizer of clopidogrel, clopidogrel was changed to prasugrel 3.75 mg daily (Japanese dose). However, the normal activity of the clopidogrel metabolizer CYP2C19 was confirmed more than 2 weeks later. Heparin-induced thrombocytopenia (HIT) antibodies were absent, and the change in platelet levels was not suspected to be due to HIT.

**Fig. 5:**
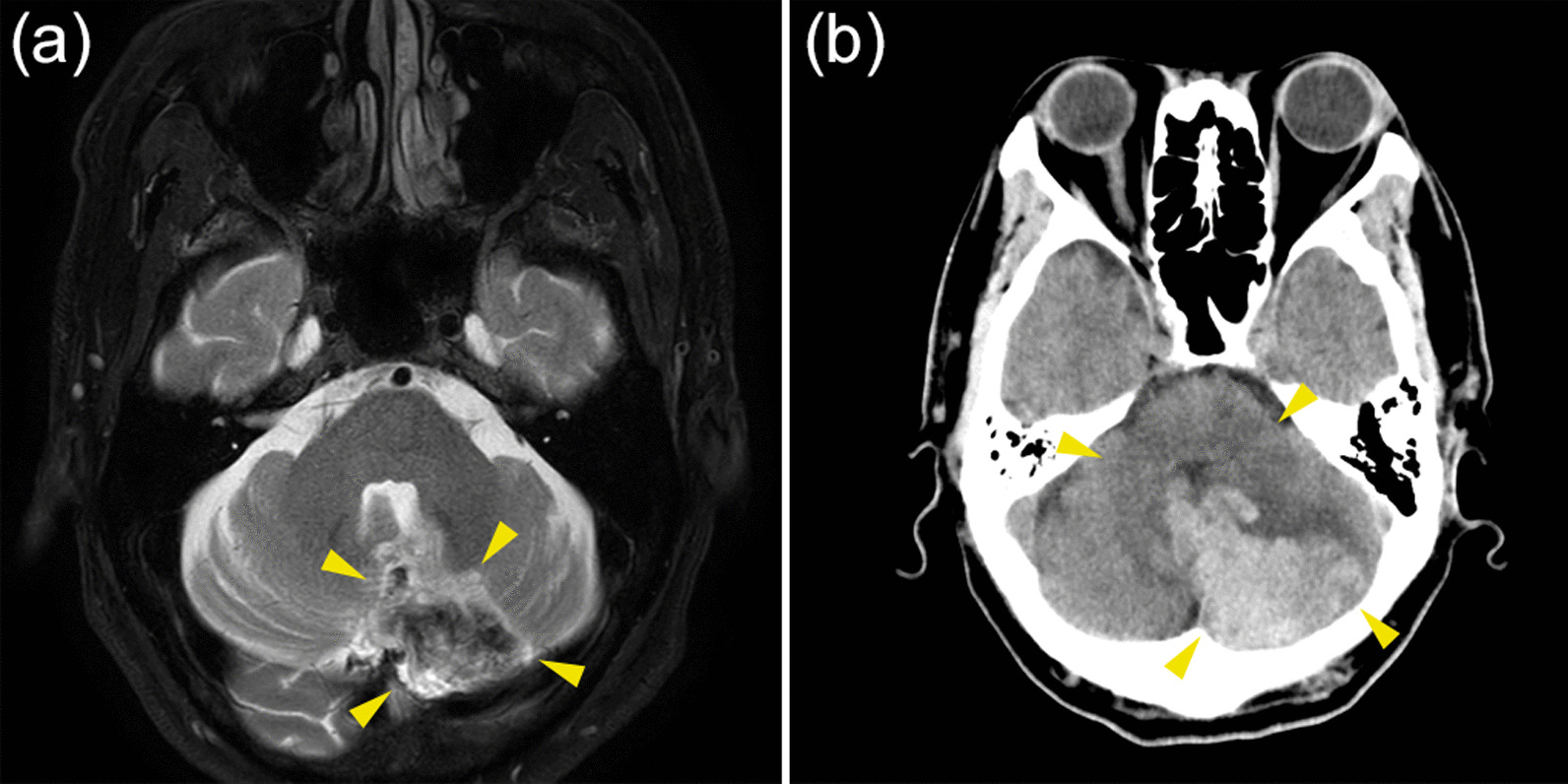
**a** T2-weighted magnetic resonance imaging (MRI) performed for diplopia 10 days after the second PCI. A heterogeneous low-signal area in the left cerebellar hemisphere and a high-signal area in its limbus (yellow arrowheads) in the posterior inferior cerebellar artery territory can be observed. Based on the symptoms and images, hemorrhagic cerebral infarction was diagnosed. **b** Three days after MRI revealed the hemorrhagic cerebral infarction, head computed tomography (CT) was performed because of the occurrence of bradykinesia. CT showed hemorrhage (high density area) and edema around it (low density area) (yellow arrowheads). The hematoma and edema have worsened and compressed the fourth ventricle

**Fig. 6:**
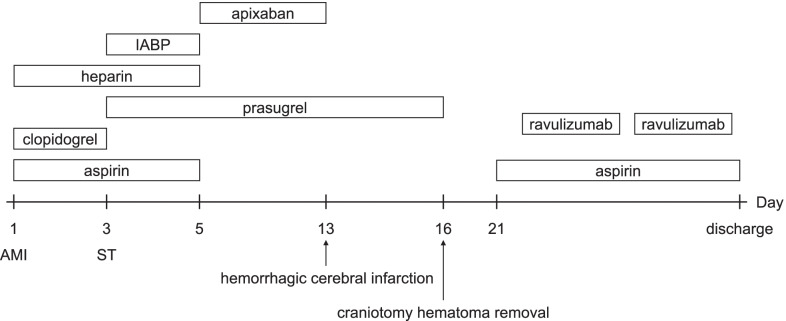
Course of treatment (mainly antithrombotic therapy) during hospitalization. Abbreviations: AMI, acute myocardial infarction; ST, stent thrombosis; IABP, intra-aortic balloon pumping

Two days after ST, IABP was discontinued, and continuous heparin was replaced with a direct oral coagulant (apixaban, at a dose of 5 mg twice daily) as a thrombotic treatment for PNH, and cardiac rehabilitation was continued. Although we planned to discontinue triple therapy during hospitalization, the patient was still on aspirin and prasugrel. Ten days after the ST, cranial magnetic resonance imaging was performed because of the development of diplopia. Hemorrhagic cerebral infarction of the left cerebellum in the territory of the posterior inferior cerebellar artery was diagnosed (Fig. 5a). After consultation with a neurologist, antithrombotic therapy was reduced from triple therapy to prasugrel alone, and computed tomography (CT) on the following day showed no obvious hematoma enlargement. However, 13 days after ST (3 days after the diagnosis of hemorrhagic cerebral infarction), the patient developed bradykinesia and underwent craniotomy hematoma removal because of an expanded hematoma observed on CT (Fig. 5b). Eighteen days after ST (5 days after craniotomy hematoma removal), antithrombotic therapy was restarted with aspirin alone because prasugrel had been discontinued owing to hematoma enlargement. The patient was discharged after rehabilitation and introduction of ravulizumab, a long-acting C5 inhibitor, for the treatment of PNH (Fig. 6). Ravulizumab was administered at an initial dose of 2700 mg, followed by 3000 mg 2 weeks later, and 3000 mg every 8 weeks thereafter. Treatment with ravulizumab injection and aspirin monotherapy as an antithrombotic therapy was continued. No evidence of thrombosis, such as new myocardial infarction or cerebral infarction, was noted for more than 1 year.

## Discussion and conclusions

The incidence of ST has been reduced because of technological advances and DAPT; however, it remains as a fatal complication that cannot be completely prevented [1]. PCI using intracoronary imaging for AMI reduces the risk of ST compared to CAG-guided PCI, although it is reported to cause ST in 0.6% of patients and is fatal in 5–45% of cases [1]. The risk varies according to the time of ST onset. In the acute to subacute phase, this includes ulcerated lesions, baseline and final thrombolysis in myocardial infarction flow of 0 or 1, younger age, diabetes mellitus requiring insulin, history of congestive heart failure, and baseline platelet count [2]. The incidence of ST in patients with diseases with thrombotic tendencies, such as PNH, is unknown.

PNH is a rare clonal hematopoietic stem cell disorder caused by an acquired mutation in the *PIGA* gene that results in the deletion of the glycosylphosphatidylinositol anchor proteins CD55 and CD59 [3]. Thrombosis is a serious complication in patients with PNH, accounting for approximately 40–67% of deaths with identifiable causes [4]. Multiple factors contribute to thrombus formation in PNH [3]. To the best of our knowledge, no study has reported the incidence of ST in patients with PNH, and case reports are very few.

In this case, the thrombotic tendency of PNH was related to AMI and thrombi in the rest of the coronary arteries. Heparin was adjusted with reference to ACT during PCI, as recommended by the guidelines [5]. Given the anticoagulant therapy for the treatment of secondary thrombi due to PNH [3, 4], continuous heparin was administered after PCI, in addition to the usual DAPT. Heparin was adjusted according to APTT values, and the dose was incrementally increased to 25,000 units/day, although the patient had chest pain and ECG changes again 2 days after PCI. CAG showed that the thrombus in the LCX had disappeared; however, ST occurred. The patient was a normal metabolizer of clopidogrel; therefore, DAPT was as effective as usual, and no antibodies or platelet changes were noted to suspect HIT. The thrombotic tendency of PNH could be related to the development of ST, and a case of repeated ST in a PNH patient with DAPT has been reported [6].When a drug-eluting stent is implanted in a patient with PNH complicated by thrombosis, antithrombotic therapy to eliminate the PNH-induced thrombus may not prevent ST.

The occurrence of thrombosis in the central nervous system of patients with PNH is reported to be 14.0% in the cerebral veins, 6.9% in the cerebral sinuses, and 4.9% in the cerebral arteries [7]. As PNH is associated with ST, anticoagulation was deemed necessary as a treatment for PNH thrombosis. After PCI for ST, the patient was switched to direct oral anticoagulants from continuous heparin, in addition to conventional DAPT. Triple therapy was only planned for use during hospitalization. Hemorrhagic cerebral infarction was identified 10 days after the second PCI. Whether cerebral infarction was catheter-related or a complication of PNH was unclear. While thrombotic complications occur in patients with PNH, hemorrhagic complications due to antithrombotic therapy should also be considered.

Effective treatments for patients with PNH include terminal complement inhibition and allogeneic hematopoietic stem cell transplantation [8]. Eculizumab is a C5 inhibitor that is effective in preventing thrombosis, and ravulizumab is a longer-acting C5 inhibitor than conventional eculizumab, reducing the burden of patient visits [8, 9]. In the present case, ravulizumab was started after the patient had stabilized following the craniotomy hematoma removal, and embolic or bleeding complications did not occur for more than 1 year. PNH clones may resolve spontaneously, and not all patients require treatment [10]. However, C5 inhibitors should be administered to patients with severe thrombosis at the earliest.

PNH is a risk factor for thrombosis, and ST occurs despite the resolution of thrombi in secondary lesions from continuous heparin administration in addition to conventional DAPT. Patients with thrombophilic diseases who undergo PCI may be prone to ST, and decisions regarding the type of antithrombotic therapy should factor the possibility of both thrombosis and bleeding. If specific treatments are available for thrombophilic diseases, they should be initiated without delay.

## Data Availability

Data could be obtained upon request to the corresponding author.

## Abbreviations

ST: Stent thrombosis
PNH: Paroxysmal nocturnal hemoglobinuria
PIGA: Phosphatidylinositol glycan class A
DAPT: Dual antiplatelet therapy
ECG: Electrocardiography
AMI: Acute myocardial infarction
CAG: Coronary angiography
RCA: Right coronary artery
LCX: Left circumflex artery
PCI: Percutaneous coronary intervention
ACT: Activated clotting time
IVUS: Intravascular ultrasound
APTT: Activated partial thromboplastin time
IABP: Intra-aortic balloon pumping
HIT: Heparin-induced thrombocytopenia
CT: Computed tomography
RAO: Right anterior oblique
LAO: Left anterior oblique
LCA: Left coronary artery
MRI: Magnetic resonance imaging
